# Intraindividual evaluation of left ventricular function with 64-slice computed tomography, biplane cineventriculography, and two- and three-dimensional transthoracic echocardiography: comparison with magnetic resonance imaging as the gold standard

**DOI:** 10.1186/1532-429X-11-S1-P233

**Published:** 2009-01-28

**Authors:** Marc Dewey, Johannes Greupner, Elke Zimmermann, Andrea Grohmann, Hans-Peter Dübel, Till Althoff, Adrian Constantin Borges, Wolfgang Rutsch, Bernd Hamm

**Affiliations:** Charite-Universitätsmedizin Berlin, Berlin, Germany

**Keywords:** Magnetic Resonance Imaging, Stroke Volume, Left Ventricular Function, Multicenter Trial, Institutional Review Board Approval

## Introduction

To intraindividually compare left ventricular function assessed with multislice computed tomography using 64 simultaneous detector rows (MSCT), biplane cineventriculography (CVG), and both 2D and 3D transthoracic echocardiography (2D and 3D Echo) with magnetic resonance imaging (MRI) as the gold standard as an ancillary single-center study of the multicenter trial "CorE-64".

## Methods

A total of 38 patients prospectively underwent MSCT (Aquilion 64, Toshiba Medical Systems, Nasu, Japan), CVG, and MRI, and 2D as well as 3D Echo. Institutional review board approval for this prospective, cardiac function, ancillary study of a multicenter trial on coronary imaging was obtained.

## Results

Regarding the ejection fraction, the agreement was significantly superior for MSCT (± 13.8%) than for CVG (± 20.4%; *P* = 0.02; F-test) and both 2D Echo (± 19.3%; *P* = 0.049; F-test) as well as 3D Echo (± 21.7%; *P* = 0.01; F-test). MSCT (56.9 ± 14.7%, *P* = 0.8), 2D Echo (56.5 ± 14.7%, *P* = 0.9, t-test) and 3D Echo (58.7 ± 16.4%, *P* = 0.34, t-test) did not significantly under- or overestimate ejection fraction in comparison to MRI (56.5 ± 16.0%), whereas CVG (60.9 ± 13.8%, P = 0.02, t-test) significantly overestimated ejection fraction. For the stroke volumes, the limits of agreement for CVG (± 56.5 ml, *P* = 0.001) and 2D and 3D Echo (± 45.4 ml and ± 49.8 ml respectively, both *P* < 0.05) were also significantly larger in comparison to MRI than for MSCT (± 31.2 ml). In comparison to the reference standard MRI, CVG but not MSCT significantly overestimated the end-diastolic volume (*P* < 0.001). In contrast, both 2D and 3D Echo significantly underestimated the end-diastolic volume (both: *P* < 0.05) in comparison to the reference standard MRI.

## Conclusion

64-slice CT appears to be significantly more accurate than CVG, 2D and 3D Echo in comparison to MRI as the reference standard. Thus, CT using 64-slice technology should allow reliable evaluation of left ventricular function in clinical practice using the same data as acquired for noninvasive coronary CT angiography.

